# Screening for inhibitor of episomal DNA identified dicumarol as a hepatitis B virus inhibitor

**DOI:** 10.1371/journal.pone.0212233

**Published:** 2019-02-19

**Authors:** Fumihiko Takeuchi, Sotaro Ikeda, Yuta Tsukamoto, Yoshikazu Iwasawa, Chen Qihao, Yukie Otakaki, Ouda Ryota, Wan-Ling Yao, Ryo Narita, Hijikata Makoto, Koichi Watashi, Takaji Wakita, Koh Takeuchi, Kazuaki Chayama, Amane Kogure, Hiroki Kato, Takashi Fujita

**Affiliations:** 1 Graduate School of Biostudies, Kyoto University, Kyoto, Japan; 2 Department of Virus Research, Institute for Frontier Life and Medical Sciences, Kyoto University, Kyoto, Japan; 3 Institute of Cardiovascular Immunology, University Hospital Bonn, Bonn, Germany; 4 Department of Immunology, Faculty of Medicine and Graduate School of Medicine, Hokkaido University, Hokkaido, Japan; 5 Centre for Structural Biology, Department of Molecular Biology and Genetics, Aarhus University, Aarhus, Denmark; 6 Department of Virology II, National Institute of Infectious Diseases, Tokyo, Japan; 7 Department of Applied Biological Science, Tokyo University of Science, Noda, Japan; 8 CREST, Japan Science and Technology Agency (JST), Saitama, Japan; 9 Molecular Profiling Research Center for Drug Discovery, National Institute of Advanced Industrial Science and Technology (AIST), Tokyo, Japan; 10 Liver Research Project Center, Hiroshima University, Hiroshima, Japan; Yonsei University, REPUBLIC OF KOREA

## Abstract

Currently, there is no available therapy to eradicate hepatitis B virus (HBV) in chronically infected individuals. This is due to the difficulty in eliminating viral covalently closed circular (ccc) DNA, which is central to the gene expression and replication of HBV. We developed an assay system for nuclear circular DNA using an integration-deficient lentiviral vector. This vector produced non-integrated circular DNA in nuclei of infected cells. We engineered this vector to encode firefly luciferase to monitor the lentiviral episome DNA. We screened 3,840 chemicals by this assay for luciferase-reducing activity and identified dicumarol, which is known to have anticoagulation activity. We confirmed that dicumarol reduced lentiviral episome DNA. Furthermore, dicumarol inhibited HBV replication in cell culture using NTCP-expressing HepG2 and primary human hepatocytes. Dicumarol reduced intracellular HBV RNA, DNA, supernatant HBV antigens and DNA. We also found that dicumarol reduced the cccDNA level in HBV infected cells, but did not affect HBV adsorption/entry. This is a novel assay system for screening inhibitors targeting nuclear cccDNA and is useful for finding new antiviral substances for HBV.

## Introduction

More than 240 million people are infected with hepatitis B virus (HBV)[1]. Although most infections in adulthood are transient, approximately 5%–10% of infected adults and over 90% of infected neonates fail to mount a sufficient immune response to clear the virus, and develop a lifelong chronic infection[2]. Every year, 0.6–1 million die from chronic hepatitis B (CHB) infection due to liver failure, cirrhosis, and hepatocellular carcinoma[3, 4]. Prophylactic vaccines have been available for hepatitis B for almost 30 years, but the overall number of chronic infections remains high[5]. Therefore, it is necessary to cure CHB infection and prevent its direct consequences[6].

HBV is a small, enveloped, double-stranded DNA virus belonging to the hepadnaviridae family[7]. Additionally, HBV is classified into eight genotypes, namely A, B, C, D, E, F, G, and H[8, 9]. Globally, HBV C and D are the most common. Covalently closed circular DNA (cccDNA) is a template for all transcription of HBV, including 3.5 kb of pregenomic RNA (pgRNA) and four viral mRNA, 3.5 kb of precore mRNA, 2.4 and 2.1 kb of surface mRNA, and 0.7 kb of X mRNA (HBx). The 3.5-kb precore mRNA contains all the open reading frames of viral proteins, but translates only the precore protein, which is further processed and secreted as the e antigen (HBeAg). pgRNA is a multifunctional transcript that encodes the viral polymerase (HBVpol) and core protein (HBc), and it serves as the template for HBV DNA synthesis. Following the binding of viral polymerase to pgRNA, the complex is packed into a nucleocapsid where polymerase-catalyzed reverse transcription yields minus-strand DNA, which is subsequently copied into plus-strand DNA to form the relaxed circular DNA (rcDNA) of the progeny. Mature nucleocapsids are then packed with viral envelope proteins to egress as virion particles[10, 11]. Therefore, cccDNA plays an essential role in the life cycle of HBV, and its elimination is necessary for curing hepatitis B[12]. Drugs currently approved for HBV treatment include interferon-alpha (IFN-α) and five nucleos(t)ide analogs, namely lamivudine (3TC), adefovir, entecavir, telbivudine, and tenofovir. Each agent has its own advantages and drawbacks[13]. For example, IFN-α is costly and poorly tolerated, and only a small proportion of patients with CHB respond to it. Nucleotide analogs can effectively block HBV replication by inhibiting reverse transcription, but drug resistance emerges after long-term treatment[14–16]. However, the complete eradication of viral cccDNA from the nuclei of infected hepatocytes cannot be achieved by the aforementioned drugs. Thus, the development of new drugs that can eliminate established cccDNA from infected hepatocytes is clinically of interest.

In a previous study to identify small molecules that inhibit HBV cccDNA, a compound library was screened using cell lines with stable expression of HBV, which induces the expression of cccDNA-dependent HBeAg as a surrogate marker for cccDNA, and two structurally related compounds that act as cccDNA formation inhibitors by blocking rcDNA deproteinization[17]. To date, cells transgenic for 1.3-fold of the HBV genome, HBV-infected primary human hepatocytes (PXB)[18], cells with stable expression of NTCP[19], and transient transfection of plasmids, including 1.3-fold of the HBV genome, have been used to reproduce HBV cccDNA generation. However, inhibition of any of the steps in the HBV replication cycle may result in cccDNA reduction. We therefore aimed to develop a screening method focusing on nuclear circular DNA inhibition.

To produce nuclear circular DNA, we used a lentivirus. After infection of a lentivirus, viral genomic RNA is reverse-transcribed to double-stranded (ds) DNA in the cytoplasm. The linear dsDNA is then transported to the nucleus where it is integrated into the genome by integrase. In the absence of integrase, the dsDNA remains linear, or is converted to the circular form by homologous recombination (HR) or non-homologous end joining (NHEJ)[20–22]. We used a mutant lentivirus defective of integrase to produce non-replicating circular DNA (lentiviral episome DNA, LeDNA) in the nucleus. In addition, we used an engineered lentivirus encoding luciferase to monitor the level of LeDNA.

We screened 3,840 chemicals using the mutant lentivirus. Our screening led to the identification of dicumarol[23], which induced a decrease in luciferase activity and LeDNA. We further demonstrated that dicumarol inhibits HBV replication and impaired HBV cccDNA levels in cells, including primary human hepatocytes. To the best of our knowledge, this is the first attempt to target cccDNA to identify a small molecule inhibitor of HBV.

## Materials and methods

### Cell culture

Human hepatocellular carcinoma (HepG2) and human embryonic kidney 293T (HEK293T) cells were maintained in Dulbecco’s modified Eagle’s medium (DMEM) supplemented with 10% fetal bovine serum (FBS), penicillin (100 U/ml), and streptomycin (100 μg/ml). Hep38.7-Tet cells were maintained in common DMEM including tetracycline (400 ng/ml) and G418 (400 μg/ml)[24–26]. HepG2 cells expressing sodium taurocholate cotransporting polypeptide (NTCP)-mCherry fusion protein, (NmcHepG2) were maintained in common DMEM including G418 (400 μg/ml). Human hepatocytes, isolated from chimeric mice with humanized liver tissue (PXB), were purchased from PhoenixBio Co. Ltd. (Hiroshima, Japan) and cultured in the previously reported dHCGM medium [10% FBS, penicillin (100 U/ml), streptomycin (100 μg/ml), 20 mM HEPES, L-proline (15 μg/ml), human recombinant insulin (0.25 μg/ml), 50 nM dexamethasone, human recombinant epidermal growth factor (5 ng/ml), 0.1 mM ascorbic acid, and 2% dimethyl sulfoxide (DMSO)][27, 28]. All cell lines were cultured at 37°C in 5% CO_2_.

### Plasmids

Plasmids were constructed as follows: pLVX-IRES-mCherry or pLVX-IRES-ZsGreen1 (Clontech) was digested using XhoI and NotI, and then firefly or renilla luciferase was inserted into the vector.

### Preparation of lentiviruses and lentivirus infection

Lentiviruses were produced via the transient transfection of HEK293T cells. Twenty-four hours after seeding at a density of 2.4 × 10^6^ cells per 100-mm dish, cells were transfected with 4 μg of pCMV-VSV-G, RSV-Rev, and pCAG-HIVgp, or with 20.5 μl of Lenti-X HTX Packing Mix (Integrase Deficient; Clontech Laboratories, Inc., Palo Alto, CA) per dish and mixed with Lipofectamine 2000 (Invitrogen) according to the manufacturer’s instructions. The following day, culture supernatants were replaced with fresh DMEM. The supernatant was filtered through a 0.45-μm filter, concentrated using Centriprep 10K to 1/20 of the original volume, and stored at −80°C. Before infection, HepG2 cells were seeded on a collagen-coated 100-mm dish at 1.5 × 10^6^ cells per dish with DMEM. Condensed lentivirus was added for 2 days with polybrene (Nacalai, 10 μg/ml), and the medium was replaced with fresh DMEM. After another day of cultivation, lentivirus-infected-HepG2 cells were seeded onto 96-well plates (2 × 10^4^ cells/well). At 24 h after incubation, cells were treated with the drug library (Pharmakon 1600 drug library, Prestwick chemical library, and Tocriscreen compound library) for 24 h, and then subjected to the dual luciferase, protein assay, or HIRT extraction.

### HBV infection

The Hep38.7-Tet cell culture medium was used as an HBV inoculum (genotype D). Hep38.7-Tet cells were seeded and cultivated for 7 days in the absence of tetracycline. The supernatant was concentrated using a Centriprep (Milipore) to 1/20 of its original volume. Cells were seeded on collagen-coated 6-well plates at 2 × 10^5^ cells per well with DMEM including G418 overnight. The medium was then replaced with dHCGM. NmcHepG2 cells were next infected with condensed HBV and polyethylene glycol 8000 (4%). The following day, the medium was replaced with dHCGM containing the test compounds.

PXB cells were infected with serum of an HBV-infected chimeric mouse (genotype C) in the presence of polyethylene glycol 8000 (4%; 5 Geq/cell) containing dHCGM. The following day, the medium was replaced with dHCGM containing the drugs.

### Enzyme-linked immunosorbent assay

Culture supernatants were analyzed using the HBV surface (HBs) ELISA (Beacle, Inc.) and HBe monoclonal ELISA (SIEMENS, Munich, Germany) kits according to the manufacturer’s instructions.

### Immunofluorescence microscopy and fluorescence imaging

Samples were fixed with 4% paraformaldehyde (PFA) for 15 min at 4°C, and then washed thrice with phosphate-buffered saline (PBS). Cells were permeabilized with 0.05% Triton X-100 in PBS for 10 min at room temperature, blocked with PBS containing 5 mg/ml BSA and 0.04% Tween 20 for 30 min, and incubated at 4°C overnight with the respective primary antibody in blocking buffer. Cells were then incubated with the secondary antibody at room temperature for 1 h. Nuclei were stained with 4,6-dimaidino-2-phenylinodole (DAPI) and cells were observed using a confocal laser microscope, TCS-SP (Leica).

### RNA and DNA detection

Lentivirus RNA and HBV DNA were extracted from supernatants using the SMITEST EX-R&D Nucleic Acid Extraction Kit (Medical & Biological Laboratories Co, Ltd, Nagoya, Japan).

Total RNA was extracted with Trizol (Invitrogen) and reverse-transcribed using the ReverTra Ace qPCR RT Kit (TOYOBO) to produce cDNA. Episomal DNA was extracted by HIRT extraction [29]. Real-time PCR (RT-PCR) for HBV core (HBc) and HBs (based on the accession number of HBV genome AB 644287) was performed using the Fast SYBER Green Master Mix (Applied Biosystems) or the THUNDERBIRD qPCR Mix (TOYOBO, Japan) according to the manufacturer’s instructions (S1 Table). The human glyceraldehyde 3-phosphate dehydrogenase (GAPDH) gene or mitochondrial DNA was used as an internal control to normalize differences in each sample.

HBV cccDNA was quantified as described elsewhere [29] by qPCR using the primer set 5′-CGTCTGTGCCTTCTCATCTGC-3′ and 5′-GCACAGCTTGGAGGCTTGAA-3′, and the probe 5′-56FAM/CTGTAGGCA/ZEN/TAAATTGGT/3IABkFQ/-3′.

### Southern blotting

The lentivirus and HBV DNA probe were synthesized by PCR using their respective primer sets. For detection of the lentivirus episome, 5 DNA fragments were synthesized using Firefly primer pairs; for HBV, 9 DNA fragments were synthesized using ccc primer pairs (S1 Table and dx.doi.org/10.17504/protocols.io.xggfjtw). The amplified DNA fragments were labeled with the Random Primer DNA Labeling Kit (Takara) according to the manufacturer’s instructions (S1 Table). All extracted DNA samples were detected according to the previously described method [28]. Signals were visualized and analyzed using a FUJIFILM BAS-5000 image scanner.

### Statistical analysis

Significance was assessed using one-way ANOVA followed by Tukey’s test for multiple comparisons. P < 0.05 was considered significant.

### DNA sequence confirmation

DNA sequences were confirmed using the BigDye Terminator v3.1 cycle Sequencing Kit (Thermo Fisher) in an ABI 3100 automated sequencer.

### Cell viability

Cell viability was examined using a WST-1 cell proliferation assay (Takara) according to the manufacturer’s instructions. For amido black staining, cells were washed with PBS and fixed with methanol. Then, 0.5% Amido Black solution was added and cells were incubated for 20 min at room temperature. After 20 min, the solution was removed and stained cells were quantified using EPSON GT-X820. Protein assay was by using Protein Assay Dye Reagent concentrate (Bio-Rad) according to the manufacturer’s instructions.

### Chemical libraries and compounds

The Pharmakon 1600 drug library (Microsourse Discovery System), Prestwick chemical library (Prestwich chemical), and Tocriscreen compound library (Tocris Bioscience) were dissolved in DMSO, and used for screening. The Prestwick chemical library was kindly gifted by Drs. Masayuki Saijo and Masayuki Shimojima (National Institute of Infectious Diseases, Tokyo, Japan). Warfarin was purchased from Funakoshi (Japan). Dicumarol and lamivudine were purchased from the Tokyo Chemical Industry (Tokyo, Japan). 7-hydroxy-4-methylcoumarin and coumarin were purchased from Nacalai.

## Results

**Fig 1:**
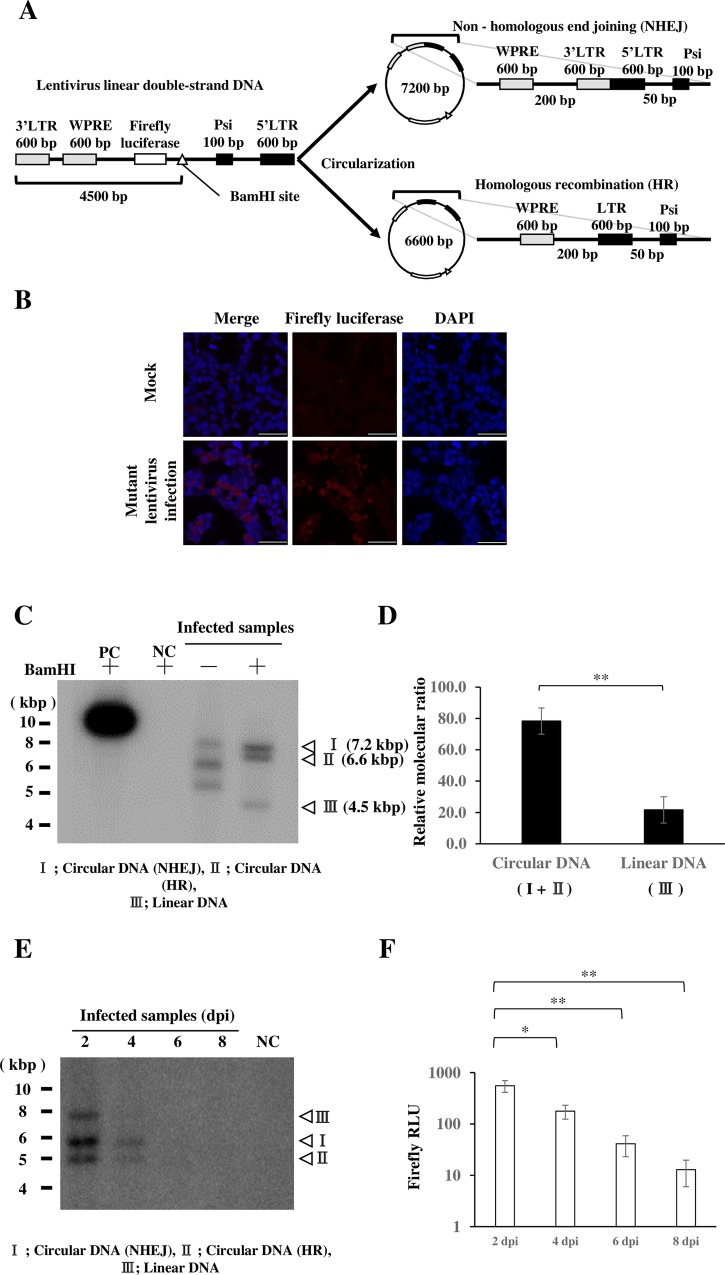
Formation of lentiviral circular DNA. (A) Formation of lentiviral circular DNA. After infection of the integration-defective lentivirus, linear dsDNA is converted into the circular form by either NHEJ or HR. (B) Lentivirus-infected HepG2 cells were analyzed by immunofluorescence using an antibody against firefly luciferase, and 26.8% of the cells were positive for luciferase expression. (C) Lentivirus-infected cells were subjected to HIRT extraction, and analyzed by Southern blotting with and without BamHI digestion. PC: positive control, lentivirus construct plasmid 1 μg, NC: negative control, mock-infected. (D) The intensity of bands on Southern blotting was measured by Multi Gauge software. I HepG2 cells were infected with the lentivirus. At 2, 4, 6, and 8 dpi, lentivirus-infected HepG2 samples were analyzed by Southern blotting. (F) At 2, 4, 6, and 8 dpi, lentivirus-infected HepG2 samples were analyzed by luciferase assay. Data are means ± SE of replicates from at least two independent experiments and were analyzed by the *t-*test: *P < 0.05, **P < 0.01.

The scheme for the LeDNA-dependent assay is illustrated in Fig 1A. Briefly, the defective lentivirus formed circular DNA by NHEJ and HR (S1 Fig). We made a lentivirus construct that encodes firefly luciferase but does not include any of the HBV sequence. After infection, 7.2 kb of linear lentiviral DNA with long terminal repeats (LTRs) at the 3′ and 5′ ends was converted into 7.2 kb of circular DNA by NHEJ or 6.6 kb of circular DNA by HR. This lentivirus has two advantages. First, only a few cell culture systems support HBV infection, but it takes at least 12 days to detect HBV replication. In contrast, lentiviruses can infect a wide range of cell lines and its gene expression can be detected before 16 h. Second, as replication of HBV takes place slowly at low levels, detection of replicated HBV DNA requires subtraction of the high background of adsorbed virion-containing genomic DNA. Lentiviruses are RNA viruses; therefore, input RNA does not interfere with intracellular LeDNA detection.

We infected HepG2 cells with the mutant lentivirus and investigated the infectivity by staining with a luciferase antibody. Based on the immunofluorescence, nearly 30% of the cells were infected with the mutant lentivirus (Fig 1B). After infection, episomal DNA was extracted by the HIRT method[30, 31]. Southern blotting detected three DNA species containing lentiviral sequences (Fig 1C). To identify the nature of these species, extracted DNA was digested with BamHI. Linearization altered the mobility, and based on the size, three forms were identified. Using the intensity and size, the molar ratio of circular (I + II) and linear (III) DNA was calculated (Fig 1D). We concluded that the lentivirus predominantly formed circular DNA. To further examine HR and NHEJ, we sequenced the 7.2- and 6.6-kbp DNA fragments. Two joined LTRs (7.2 kbp), and one LTR flanked by WPRE and Psi (6.6 kbp) were confirmed (S2 Fig).

As the episomal lentivirus DNA lacked a replication origin, its level was expected to decline as the cells proliferated. To confirm this, lentiviral DNA was monitored. At 2, 4, 6, and 8 dpi, cells were harvested to extract HIRT DNA. Southern blotting revealed that episomal DNA levels markedly decreased over time (Fig 1E). Luciferase expression was similarly monitored and it decreased exponentially (Fig 1F). On the other hand, LeDNA was maintained (half life >9 days) when cell growth was arrested (S3 Fig).

**Fig 2:**
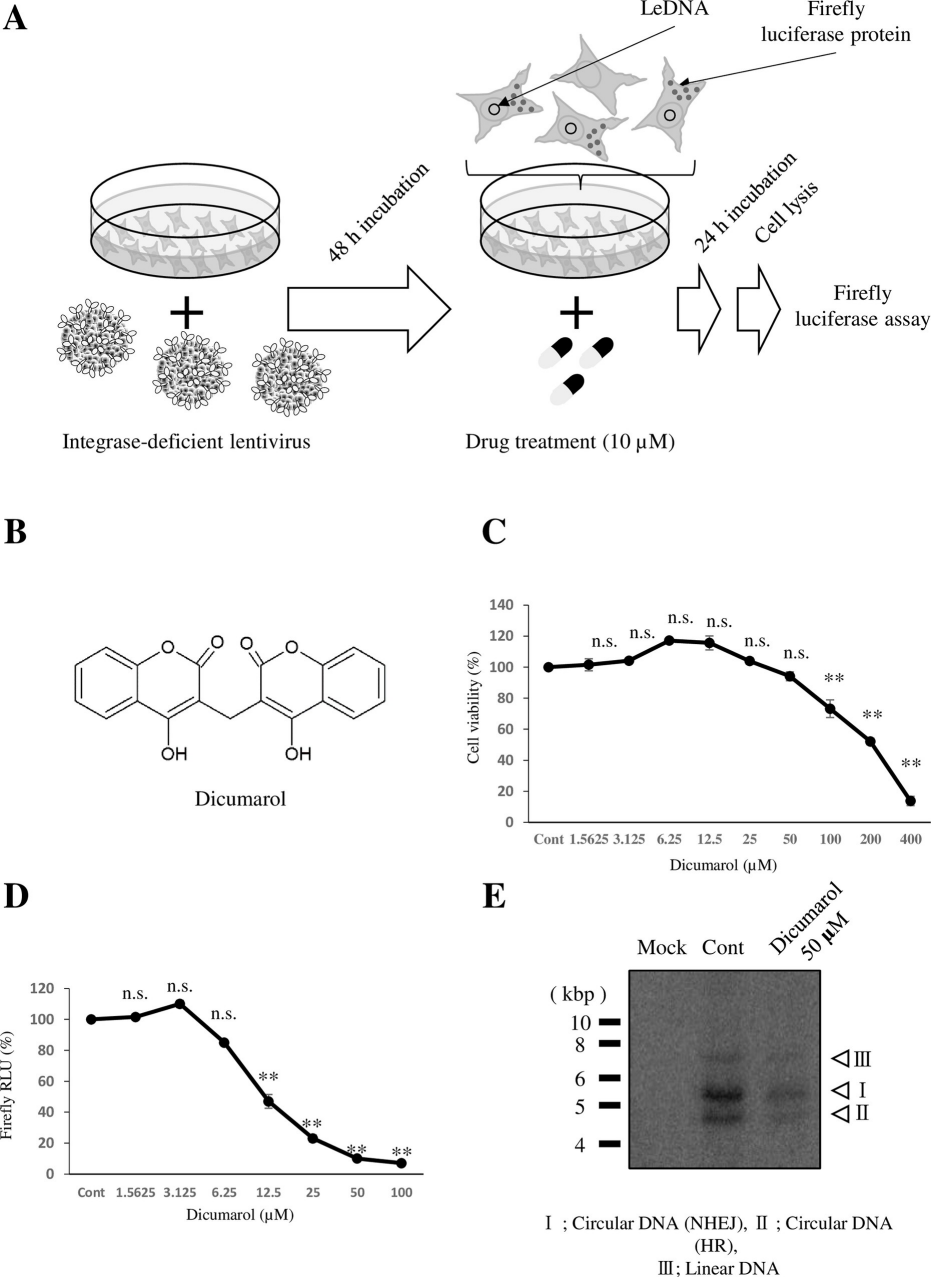
Dicumarol inhibits LeDNA. HepG2 cells were infected with the integrase-deficient lentivirus for 2 days and treated with chemicals (10 μM) for 1 day. Cells were then subjected to assays for luciferase and LeDNA (A). (B) The chemical structure of dicumarol. Lentivirus-infected HepG2 cells were treated with the indicated concentrations of dicumarol. After 24 h, samples were collected and analyzed for luciferase (C) and cell toxicity (D). LeDNA was analyzed by Southern blottiI(E). Data are means ± SE of replicates from at least two independent experiments and were analyzed by the *t-*test: *P < 0.05, **P < 0.01.

We screened 3,840 chemicals using luciferase activity as a readout. Cells were infected with the lentivirus and treated with chemicals, as shown in Fig 2A. At the end of the cultivation, cells were harvested for luciferase activity and protein assay. Primary screening was performed using 10-drug mix and repeated at least 3 times. 10 of 10-drug mix exhibited enhanced luciferase activity, which were not analyzed further. 12 of 10-drug mix, which exhibited reduced luciferase activity, were further examined for each chemical as secondary screening. We identified 12 candidate inhibitors. We focused on one of the candidates, dicumarol, which exhibited lowest effective concentration. Dicumarol is known to possess anticoagulant activity (Fig 2B). Dicumarol exhibited cell toxicity at concentrations higher than 100 μM (CC50 225 μM) (Fig 2C); however, as strong luciferase inhibition was observed at 25–50 μM (EC50 12 μM). Because the therapeutic index (TI; EC50/CC50) was 0.05, this inhibition was not only due to toxicity (Fig 2D). Southern blotting revealed that dicumarol reduced LeDNA at 50 μM (Fig 2E). These results demonstrated that dicumarol can reduce the LeDNA level.

**Fig 3:**
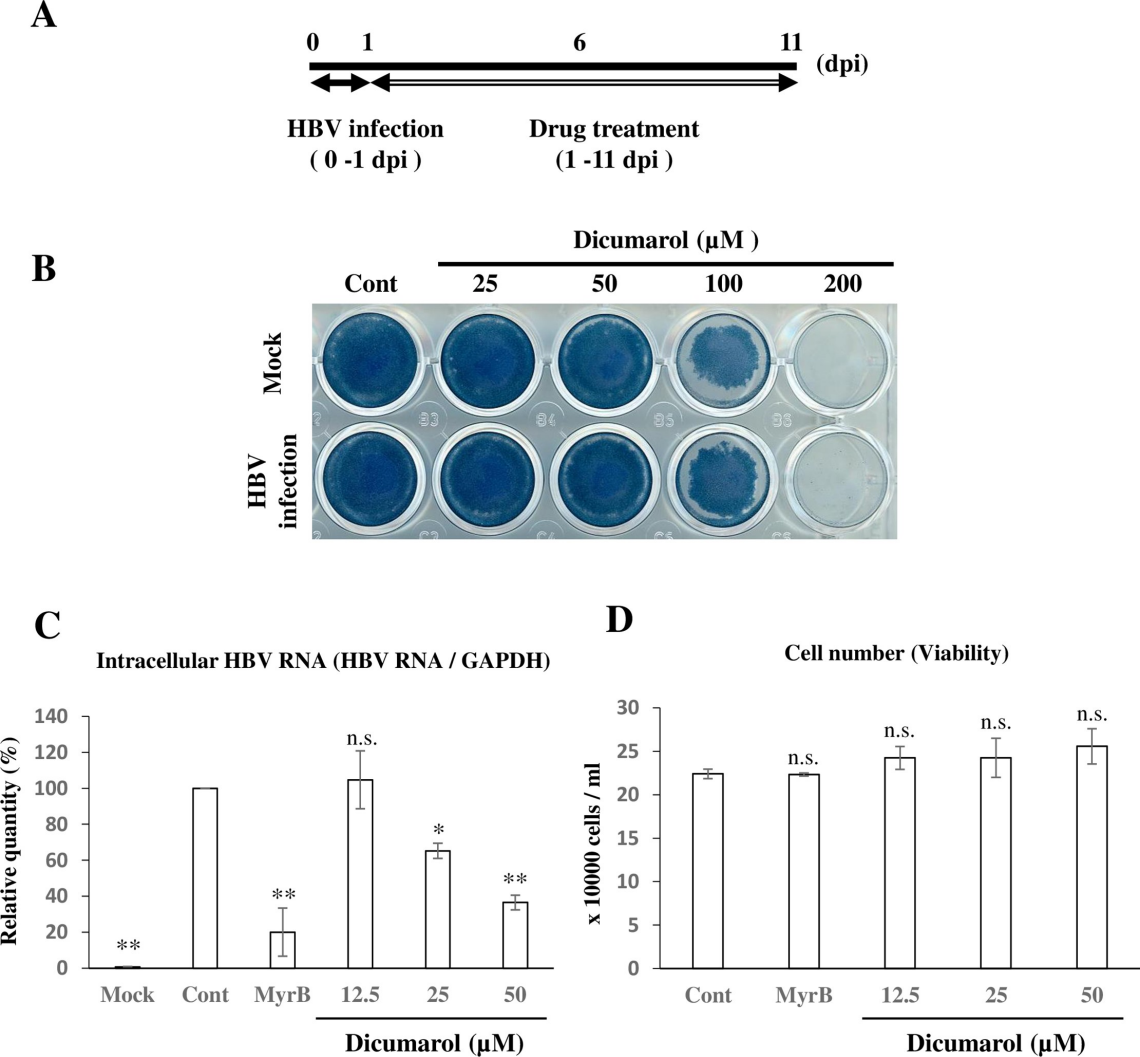
Dicumarol inhibits HBV replication in NmcHepG2 cells. (A) Scheme for infection and drug treatment. NmcHepG2 cells were infected with HBV in the absence or presence of MyrB at 0–1 dpi. At 1 dpi, the virus-containing medium was removed and a dicumarol-containing medium was added. Every 2 days, the medium was changed and samples were collected at 11 dpi. (B) Cell toxicity was examined by amido black staining. (C) At 11 dpi, RNA was quantified by qPCR. Mock: uninfected, MyrB: Myrcludex B, cont: mutant MyrB. (D) Cell viability was confirmed by counting the cell number. Data are means ± SE of replicates from three independent experiments and were analyzed by the *t-*test: *P < 0.05, **P < 0.01.

Next, we examined anti-HBV activity of dicumarol. NmcHepG2 cells were infected with genotype D HBV derived from Hep38.7-tet cells (Materials and Methods, Fig 3A). We examined drug toxicity by amido black staining. To distinguish between non-specific adsorption of HBV inoculum and intracellular HBV replication, Myrcludex B peptide (MyrB) was used. MyrB, which is a preS1-derived peptide and competitive inhibitor of HBV entry, inhibits NTCP-mediated infection [32], whereas its mutant is incapable of blocking HBV entry. Dicumarol exhibited CC50 120 μM, but very little toxicity was observed at lower concentrations (Fig 3B). According to qPCR, dicumarol inhibited HBV RNA expression in a dose-dependent manner with EC50 40 μM and TI was 0.33 (Fig 3C). Under these conditions, dicumarol did not exhibit toxicity (Fig 3D).

**Fig 4:**
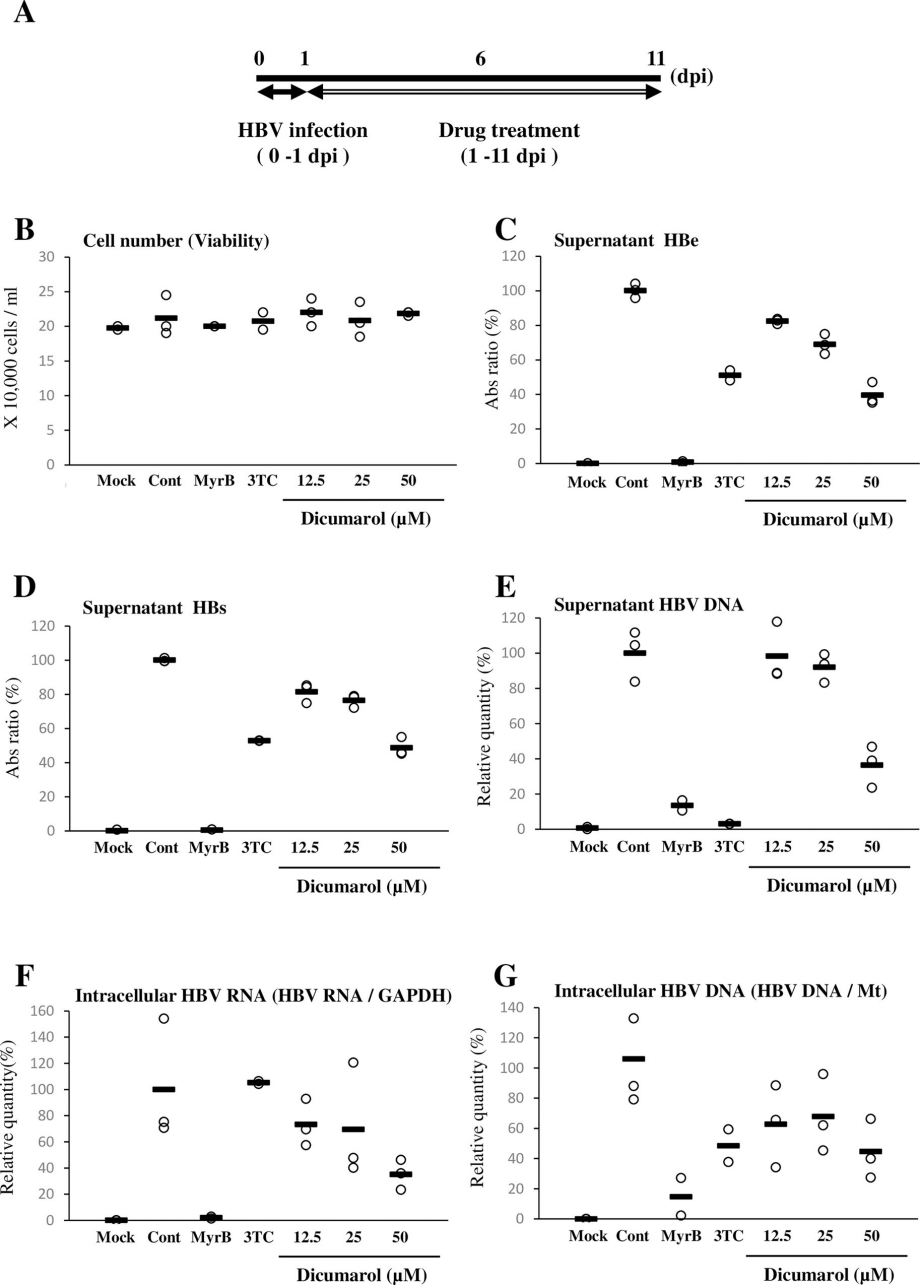
Dicumarol inhibits HBV replication in HBV-infected primary human hepatocytes. (A) Experimental scheme. PXB cells were infected with HBV in the absence or presence of MyrB at 0–1 dpi. At 1 dpi, the virus-containing medium was removed and a dicumarol-containing medium was added. Every 2 days, the medium was changed and samples were collected at 11 dpi. 3TC: 500 nM lamivudine, which is a reverse transcriptase inhibitor. (B) Cell viability was examined by counting the trypan blue-negative cell number. (C) HBe in the culture supernatant. (D) HBs in the culture supernaIt. (E) The DNA in the supernatant was extracted and quantified by qPCR. (F) Intracellular HBV RNA was quantified by qPCR. (G) Intracellular HBV DNA was quantified by qPCR. Data are means ± SD of at least duplicate measurements.

Next, we examined the effects of dicumarol on HBV replication in primary human hepatocytes, PXB (Fig 4A). Here, a known HBV inhibitor lamivudine (3TC), a reverse transcriptase inhibitor was used as positive control. 3TC inhibits the HBV DNA synthesis because HBV DNA synthesizes DNA solely by reverse transcription. Thus supernatant DNA level is strongly reduced (Fig 4E). Whereas, HBV RNA level (as determined by HBs PCR primer) level was not significantly affected (Fig 4F). Similarly extracellular secretion of HBs and HBe was only partially inhibited (Fig 4C and 4D). Dicumarol did not exhibit toxicity within the range of concentrations used (Fig 4B). On the other hand, dicumarol inhibited the release of Hbe (Fig 4C), HBs (Fig 4D), and HBV DNA (Fig 4E) in a dose-dependent manner. Similarly, intracellular accumulation of HBV RNA (Fig 4F) and DNA (Fig 4G) was inhibited. These findings demonstrated that dicumarol has anti-HBV activity.

**Fig 5:**
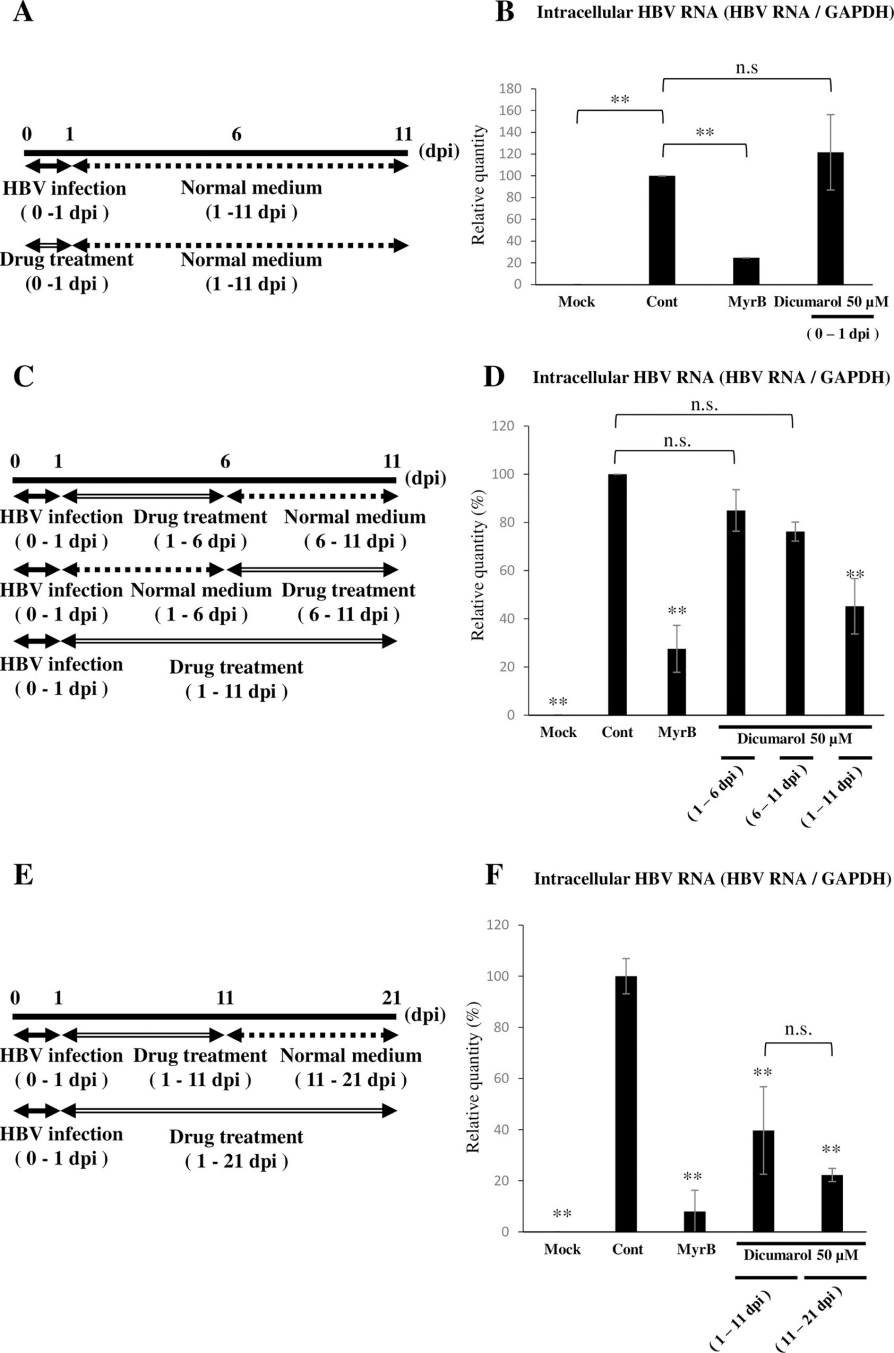
Dicumarol did not inhibit HBV adsorption/entry. NmcHepG2 cells were infected with HBV in the absence or presence of dicumarol or MyrB at 0–1 dpi (A). (B) At 11 dpi, total cell RNA was isolated and quantified for HBV RNA. Next, cells were treated with dicumarol during HBV replication, as shown in C. As a reference, cells were untreated (cont) or treated with MyrB during HBV adsorption/entry (MyrB). (D) At 11 dpi, RNA was extracted and quantified for HBV RNA. To evaluate the effects of dicumarol during long-term HBV infection (21 days), cells were infected and treated with HBV, as shown in E. (F) At 21 dpi, RNA was extracted. HBV RNA levels relative to the control HBV replication level are shown. Controls are as in D. Data are means ± SE of replicates from three independent experiments and were analyzed by the *t-*test: *P < 0.05, **P < 0.01.

To address whether dicumarol affects the adsorption/entry of HBV, cells were mock-treated or treated with dicumarol at 0–1 dpi (Fig 5A). HBV RNA expression was not affected by dicumarol in the adsorption/entry step, contrary to the entry inhibitor MyrB (Fig 5B). Next, cells were treated with dicumarol after entry, as shown in Fig 5C. HBV replication in cells treated with dicumarol at 1–6 or 6–11 dpi was not significantly inhibited. However continuous treatment (1–11 dpi) significantly impaired HBV replication (Fig 5D), suggesting that its continuous presence is necessary for the complete block of HBV replication. We further found that dicumarol inhibited HBV infection during long-term cultivation (1–21 dpi) (Fig 5E and 5F).

**Fig 6:**
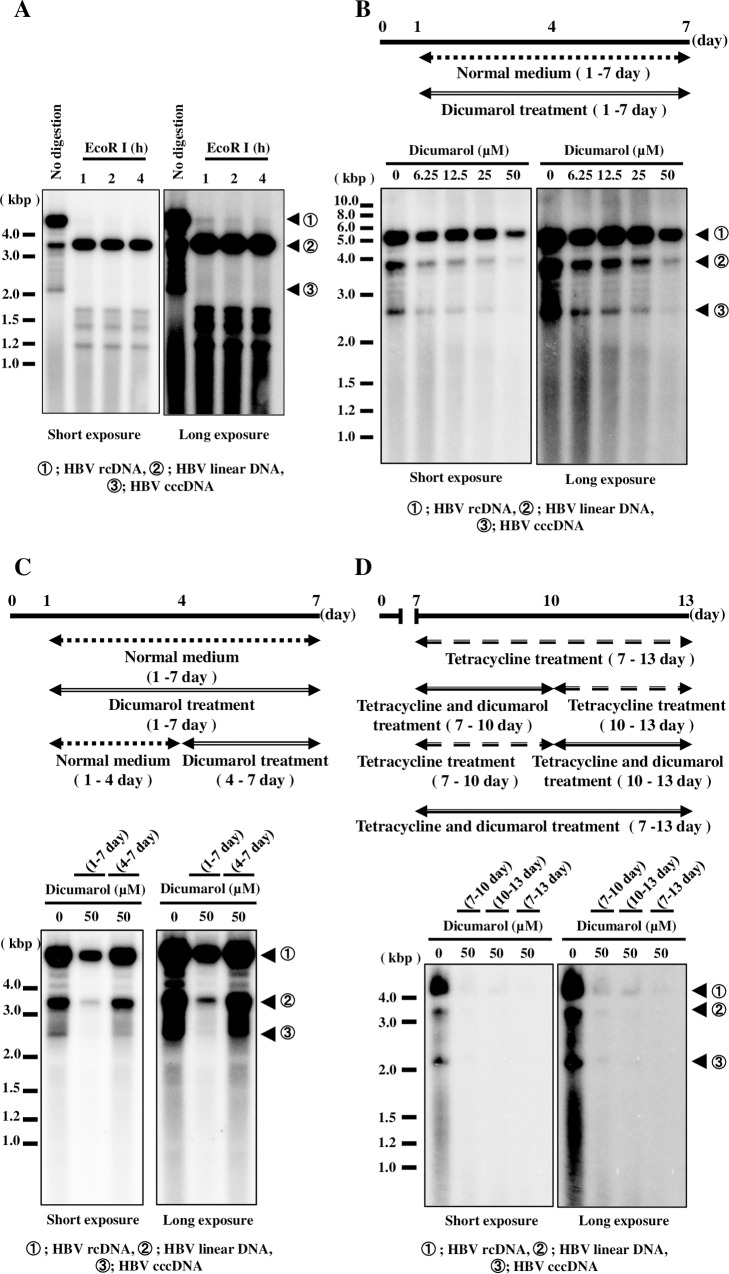
Dicumarol inhibits HBV cccDNA. HBV expression was induced in Hep38.7 cells by depleting tetracycline in the common DMEM including G418 medium for 7 days. (A) HIRT DNA was extracted and analyzed by Southern blotting. The DNA was undigested or digested with EcoRI for 1, 2, or 3 h. The positions of rcDNA, linear DNA, and cccDNA are indicated. (B) Cells were treated with increasing amounts of dicumarol as indicated. HIRT DNA (undigested) was subjected to Southern blotting. (C) Cells were treated with dicumarol as shown. HIRT DNA was extracted at 7 day and subjected to Southern blotting. (D) At first, Hep38.7 cells were cultured in common DMEM including G418 medium without tetracycline at 0–7 day. At 7day, cell medium was replaced fresh DMEM including tetracycline in the absence (dicumarol 0 μM, 10–13 day dicumarol 50 μM) or presence (7–10 day dicumarol 50 μM, 7–13 day dicumarol 50 μM) of dicumarol. At 10 day, cell medium was replaced fresh DMEM including tetracycline in the absence (dicumarol 0 μM, 7–10 day dicumarol 50 μM) or presence (10–13 day dicumarol 50 μM, 7–13 day dicumarol 50 μM) of dicumarol. Finally, HIRT DNA was collected at 13 day. Data are from one representative of at least two independent experiments.

We next investigated the effects of dicumarol on HBV cccDNA. We used Hep38.7 cells expressing HBV from the transgenic HBV genome. This cell line expresses HBV DNA upon removal of tetracycline in the culture medium. By Southern blotting analysis, 3 episomal HBV DNA species were detected (Fig 6A, no digestion). After digestion with EcoRI, which cleaves once in the type D HBV genome, these bands became 3.2 kbp, confirming these bands to be episomal HBV DNA. From their mobility on agarose gel, we identified cccDNA as the approximately 2.1-kbp band, as estimated by molecular marker positions (Fig 6A). Dicumarol treatment for 6 days markedly reduced HBV cccDNA in a dose-dependent manner, whereas the level of HBV rcDNA was minimally attenuated (Fig 6B). Consistent with this observation, intracellular HBc accumulation was not suppressed by dicumarol (S4 Fig). When cells were treated with dicumarol during the last 3 days of HBV replication (Fig 6C, 4–7 day), inhibition of HBV cccDNA was less efficient compared with continuous dicumarol treatment (1–7 day) (Fig 6C). To examine the effect of dicumarol on the decay of HBV cccDNA, Hep38.7 cells were cultivated in the absence of tetracycline (0–7 day, HBV synthesis on), then tetracycline was added to block HBV synthesis from the transgene (7–13 day). The cells were further treated with dicumarol as shown in Fig 6D in the presence of tetracycline. HBV cccDNA was maintained after 7 days cultivation in the presence of tetracycline (Fig 6D, 0 μM dicumarol). However treatment with dicumarol strongly inhibited HBV DNA maintenance including cccDNA (Fig 6D, all time course of 50 μM dicumarol). The results suggest that dicumarol accelerated decay of HBV cccDNA.

**Fig 7:**
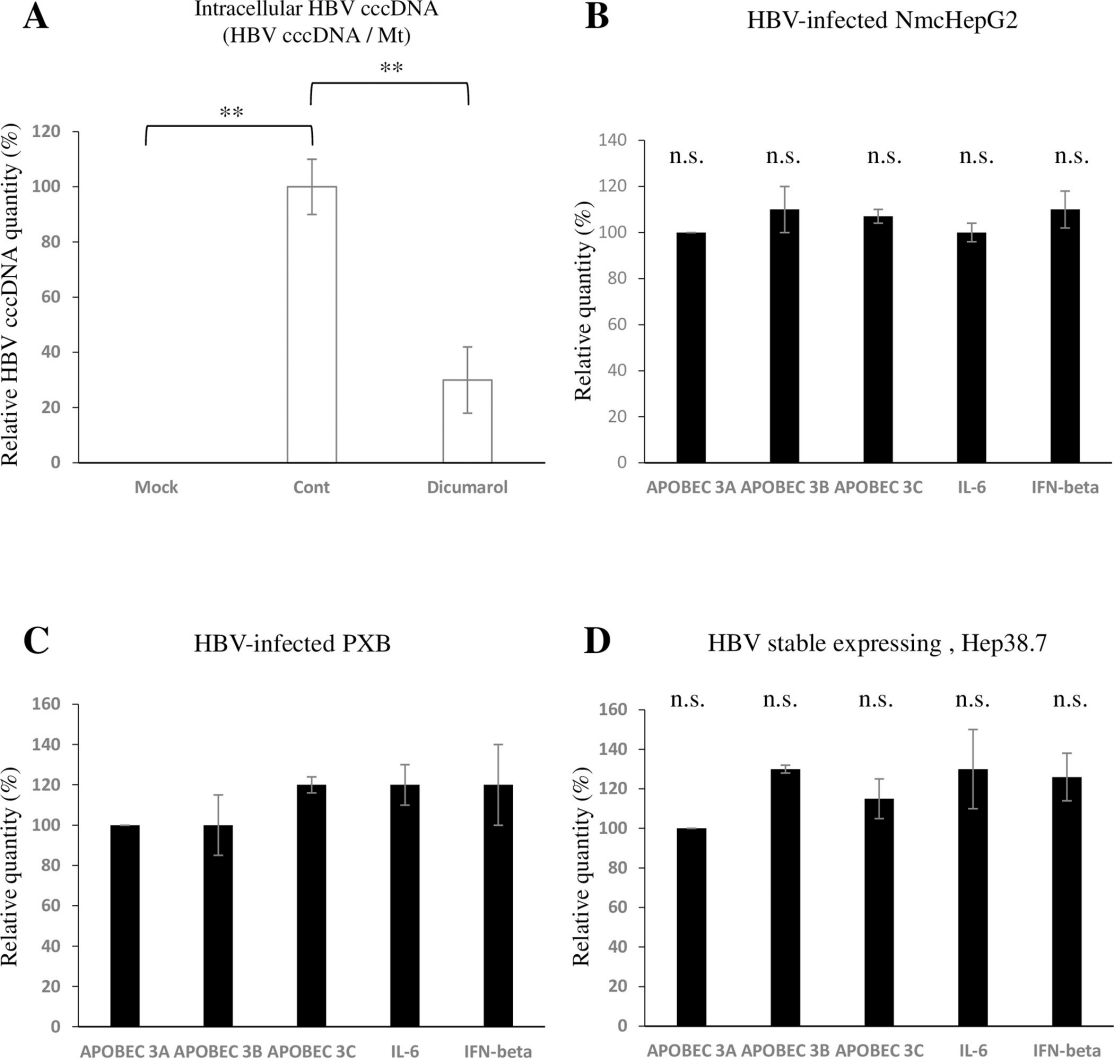
Dicumarol did not induce APOBEC, NF-κB, or ISG expression. (A) NmcHepG2 cells were infected with HBV (genotype D) and cultured for 14 days, and cccDNA was quantified by specific qPCR (Materials and Methods). (B) NmcHepG2 cells were infected with HBV in the absence or presence of 50 μM of dicumarol for 14 days, and mRNA levels of APOBEC 3A, 3B, 3C IL-6, and IFN-β were measured by qPCR. mRNA levels relative to control mRNA are indicated. (C) RNA extracted from HBV-infected PXB cells for 14 days was analyzed as in B. (D) HBV expression was induced in Hep38.7 cells for 6 days and mRNA expression was analyzed as in B. Data are means ± SE of replicates from at least two independent experiments and were analyzed by the *t-*test: *P < 0.05, **P < 0.01. n.s.: not significant.

To evaluate the inhibition of cccDNA by dicumarol in HBV infection, instead of using a transgenic model, NmcHepG2 cells were infected with HBV. As HBV cccDNA levels were much lower than those in Hep38.7 cells, we adopted quantitative PCR for cccDNA detection[29]. As shown in Fig 7A, dicumarol reduced the cccDNA level during HBV infection.

The report that human APOBEC3A and B proteins help to eradicate HBV cccDNA[33] prompted us to examine if APOBEC3 genes are up-regulated by dicumarol. Dicumarol treatment of NmcHepG2 cells did not influence the expression of APOBEC 3A, 3B, 3C, IL-6, or IFN-β genes (Fig 7B). Similar results were obtained for primary hepatocytes (PXB, Fig 7C) and Hep38.7 cells (Fig 7D).

## Discussion

We report here a unique assay for non-replicating episomal DNA. Using this assay, we screened a chemical library and identified dicumarol as an inhibitor of LeDNA, demonstrating that the assay functioned as expected (Fig 2). Although the assay had little to do with the HBV life cycle, dicumarol inhibited HBV replication (Figs 3–5). It is important to determine which stage of the HBV life cycle is affected by dicumarol because blockade of any step of the HBV life cycle may reduce HBV cccDNA. Dicumarol significantly inhibited intracellular HBV RNA, DNA, supernatant HBV DNA, Hbe, and HBs during HBV infection (Fig 4). However, in cells expressing HBV from the 1.3-fold HBV genome, intracellular HBc expression was minimally affected (S4 Fig), whereas cccDNA expression was markedly reduced (Fig 6B and 6C). These results suggested that dicumarol does not influence HBV transcription via the host RNA polymerase, but specifically inhibits cccDNA levels. LeDNA and HBV cccDNA both exist as circular dsDNA in the nucleus and have no replication origin; therefore, the potential target of dicumarol may function in the process of their formation or stability. In the case of LeDNA, it is produced after initial lentiviral infection, but does not renew. On the other hand, HBV cccDNA decay was accelerated by dicumarol (Fig 6D). It is an interesting question if dicumarol treatment can totally eliminate HBV cccDNA in the infected hepatocytes, because drug-resistant low copy number of HBV cccDNA potentially reconstitutes HBV replication cycle after drug removal. Additionally, dicumarol treatment induced the reduction of HBV linear DNA, rcDNA, and cccDNA (Fig 6B and 6D). There is the possibility that dicumarol inhibits not only HBV cccDNA but also all HBV DNA forms. These issues remained to be answered by long term (>year) in vitro and in vivo examination with HBV infection. Concerning the mechanism of cccDNA eradication, it is possible that host cells facilitate the decay of non-chromosomal small DNA and dicumarol can accelerate this process. APOBEC3 proteins, including human APOBEC3A and B, were reported to play a role in editing HBV cccDNA and its subsequent decay [33]. Expression of APOBEC3A and B is up-regulated by IFN-α and lymphotoxin-β, respectively [33]. However, dicumarol did not up-regulate APOBEC3 or IFN-α expression in HBV-infected cells (Fig 7), suggesting that dicumarol does not inhibit cccDNA through APOBEC3 proteins. Dicumarol does not directly inhibit the blood coagulation cascade, but inhibits vitamin K epoxide reductase (VKOR)[34]. Warfarin, a more potent and specific inhibitor of VKOR, and dicumarol-related compounds (S5 Fig) did not inhibit cccDNA, suggesting a possibility that dicumarol blocks other reductase(s).

In summary, the assay system we developed is effective for identifying inhibitors of episomal DNA. Our screening is still on-going, and the identification of additional inhibitors will provide insights into the host mechanism of eradicating non-chromosomal small DNA. Whether viral DNA with a replication origin is controlled by this mechanism is also of interest.

## Supporting information

S1 Table Primer sets. (TIF)

S1 Fig Formation of HBV cccDNA and LeDNA. Schematic representation of the formation of HBV cccDNA and LeDNA in infected cells.
(TIF)

S2 Fig Structure of circular lentiviral DNA resulting from HR and NHEJ. HepG2 cells were infected with the integrase-defective lentivirus, and nuclear episome DNA was extracted and sequenced. Two types of sequences were obtained. The sequences on the left and right correspond to circular DNA produced by HR and NHEJ, respectively. The sequence in orange represents LTR, that in green represents WPRE, and that in blue represents Psi.
(TIF)

S3 Fig Maintenance of circular lentiviral DNA in the proliferation stopped. HepG2 cells were cultured in dHCGM medium and infected with the integrase-deficient lentivirus for 2 days. At 3, 6, and 9 dpi, lentivirus-infected HepG2 samples were analyzed by Southern blotting.
(TIF)

S4 Fig Dicumarol did not inhibit HBc accumulation in Hep38.7-Tet cells transgenic for the 1.3-fold HBV genome. Hep38.7-Tet cells were cultured in the absence (Cont) or presence of dicumarol or tetracycline as indicated, and intracellular HBc was detected by immunostaining or DAPI staining.
(TIF)

S5 Fig Dicumarol-related compounds did not inhibit HBV replication. Dicumarol-related compounds, coumarin, warfarin, and 7-hydroxy-4-methycoumarin, were tested for anti-HBV activity. (A) Structures of dicumarol-related compounds. Cells were infected with HBV, and treated with dicumarol or dicumarol-related compounds for 1–11 dpi. (B) HBV RNA levels were measured at 11 dpi. Data are means ± SE of replicates from three independent experiments, and significance was analyzed by the *t-*test: *P < 0.05, **P < 0.01. n.s.: not significant
(TIF)
