# Supplementary figures and images for: Screening for inhibitor of episomal DNA identified dicumarol as a hepatitis B virus inhibitor
Source: PLoS One. 2019 Feb 19;14(2):e0212233. doi: 10.1371/journal.pone.0212233 (PMC6380541; doi:10.1371/journal.pone.0212233)

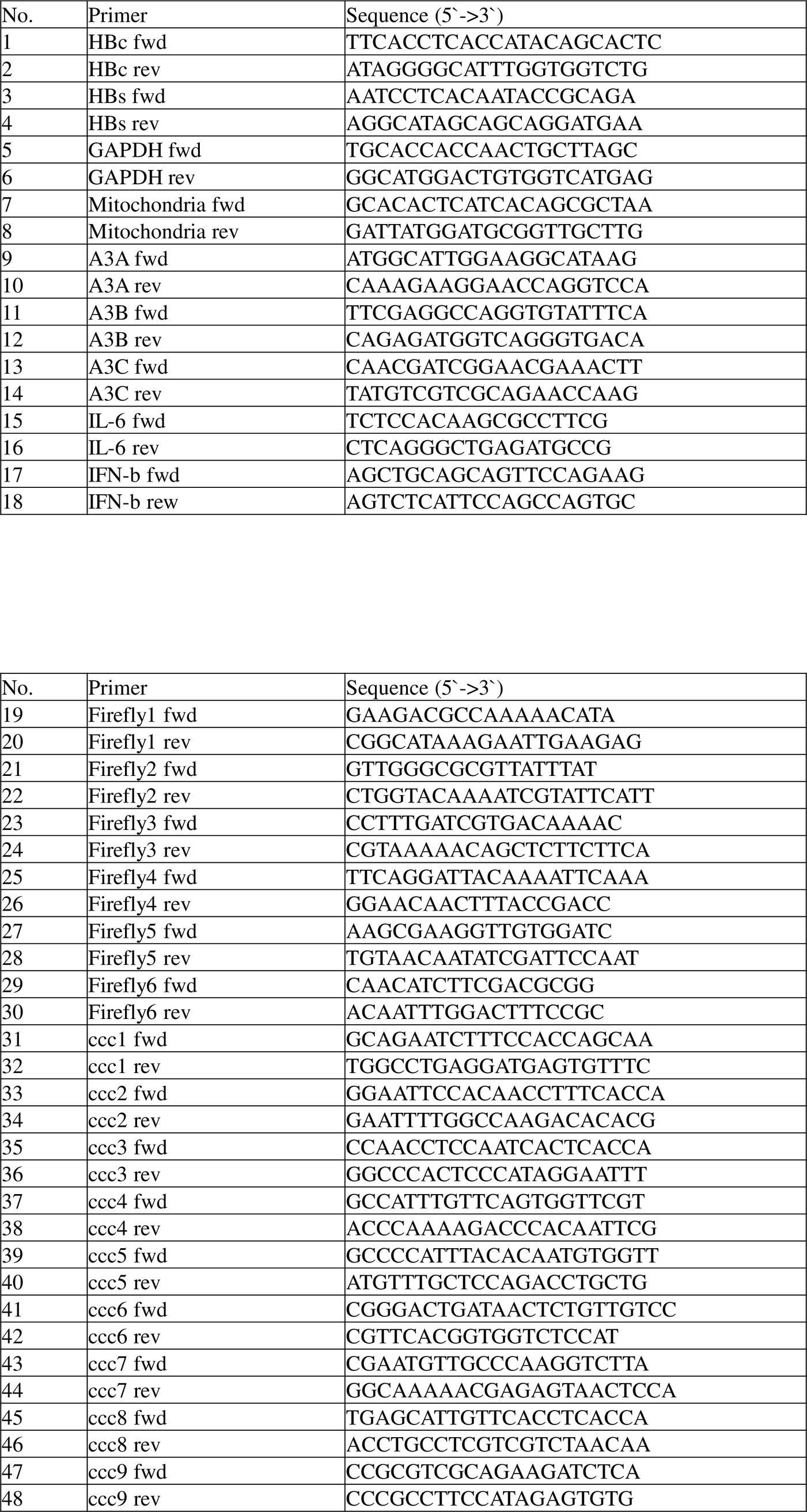

Supplement: S1 Table — (TIF) [file pone.0212233.s001.tif]

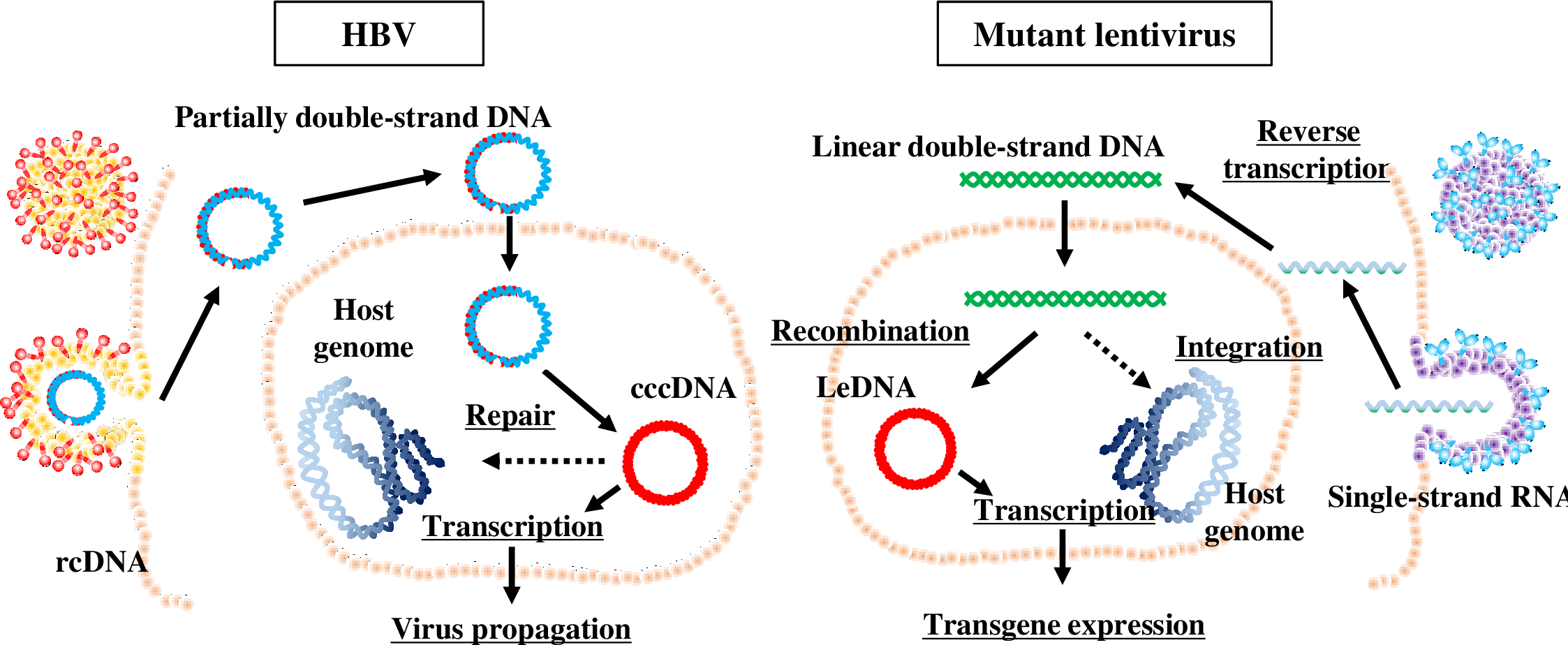

Supplement: S1 Fig — Schematic representation of the formation of HBV cccDNA and LeDNA in infected cells. (TIF) [file pone.0212233.s002.tif]

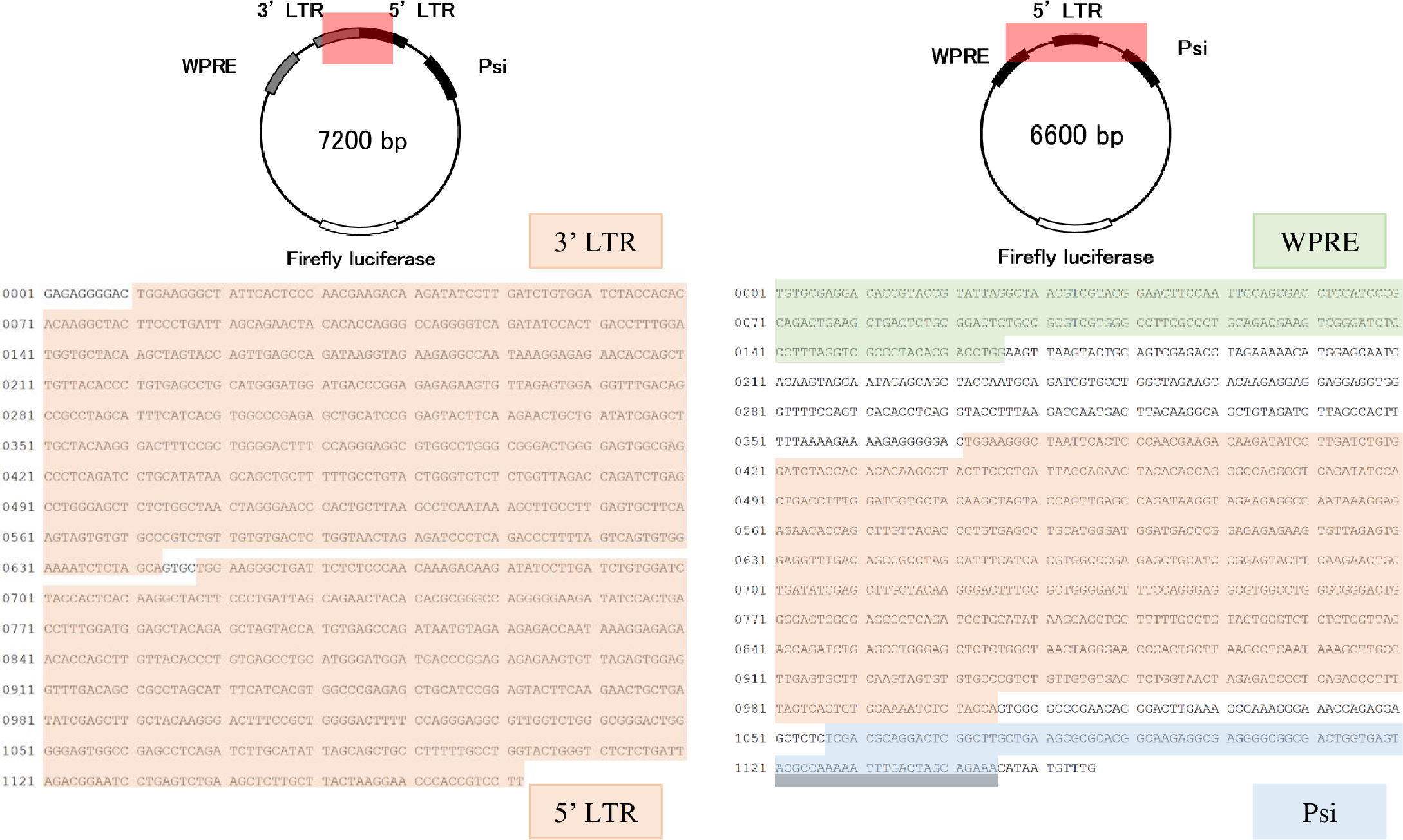

Supplement: S2 Fig — HepG2 cells were infected with the integrase-defective lentivirus, and nuclear episome DNA was extracted and sequenced. Two types of sequences were obtained. The sequences on the left and right correspond to circular DNA produced by HR and NHEJ, respectively. The sequence in orange represents LTR, that in green represents WPRE, and that in blue represents Psi. (TIF) [file pone.0212233.s003.tif]

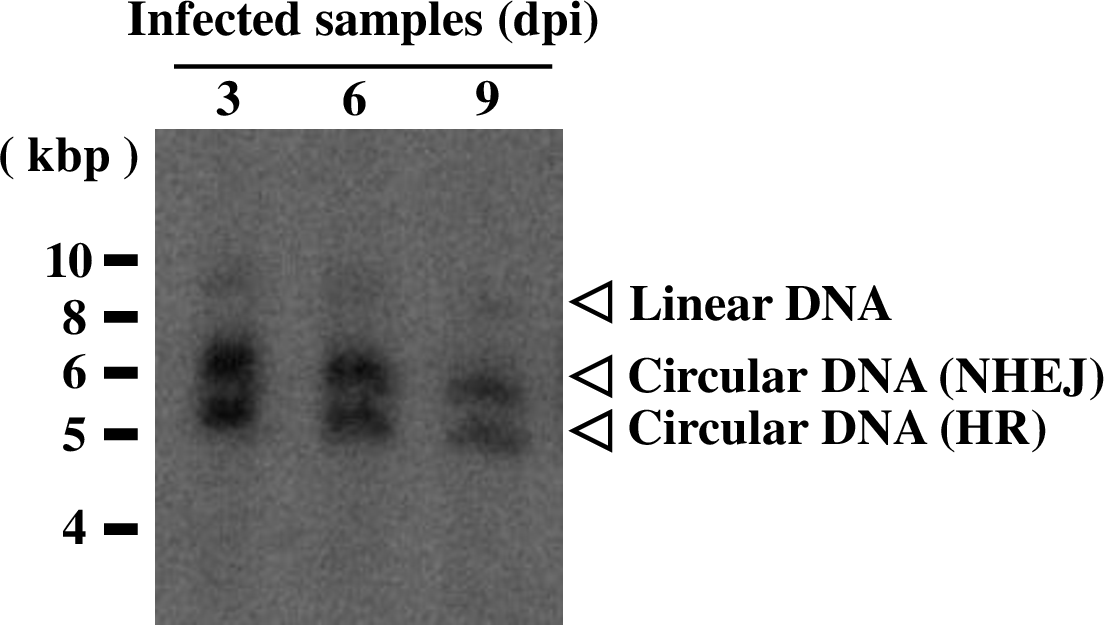

Supplement: S3 Fig — HepG2 cells were cultured in dHCGM medium and infected with the integrase-deficient lentivirus for 2 days. At 3, 6, and 9 dpi, lentivirus-infected HepG2 samples were analyzed by Southern blotting. (TIF) [file pone.0212233.s004.tif]

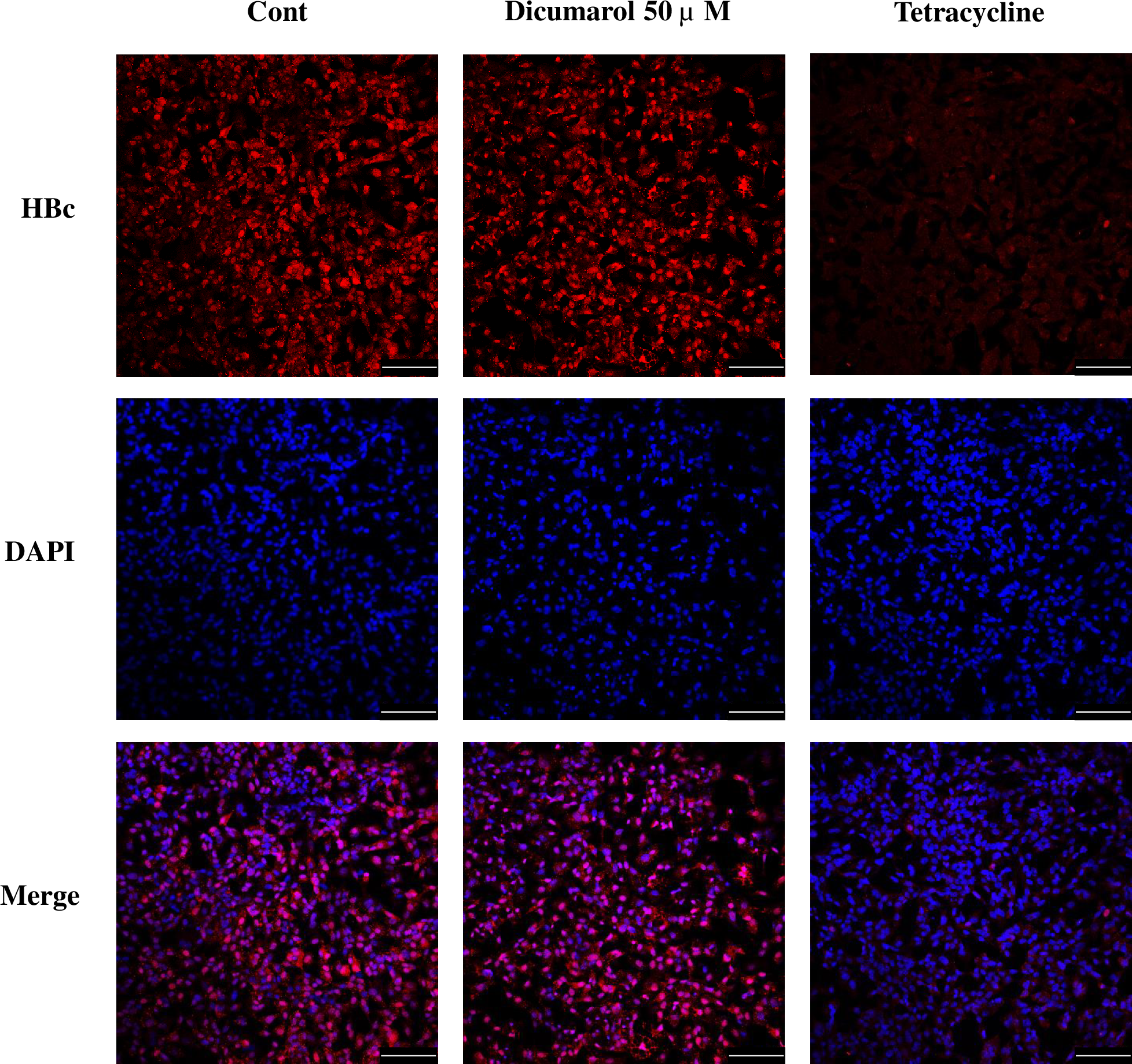

Supplement: S4 Fig — Hep38.7-Tet cells were cultured in the absence (Cont) or presence of dicumarol or tetracycline as indicated, and intracellular HBc was detected by immunostaining or DAPI staining. (TIF) [file pone.0212233.s005.tif]

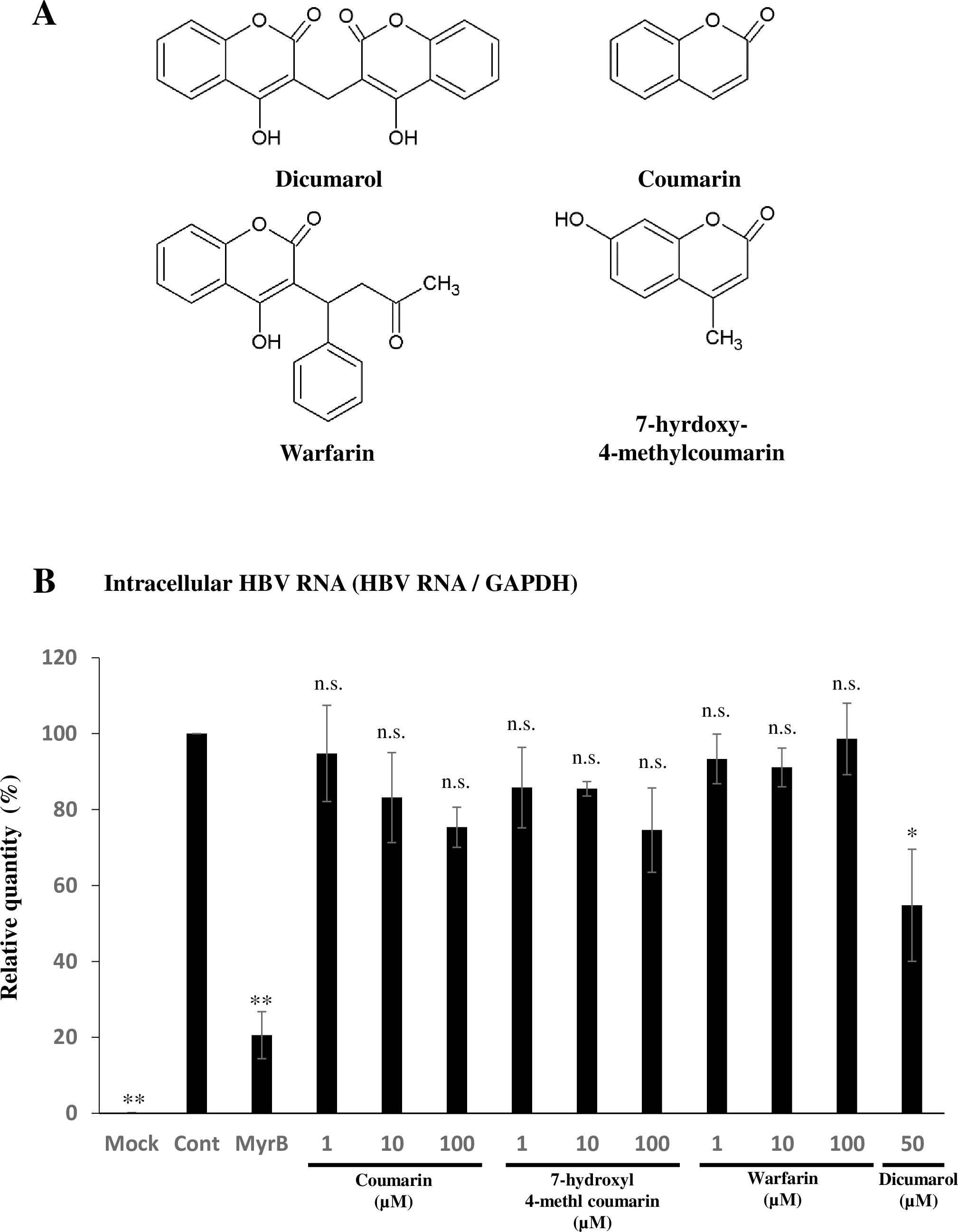

Supplement: S5 Fig — Dicumarol-related compounds, coumarin, warfarin, and 7-hydroxy-4-methycoumarin, were tested for anti-HBV activity. (A) Structures of dicumarol-related compounds. Cells were infected with HBV, and treated with dicumarol or dicumarol-related compounds for 1–11 dpi. (B) HBV RNA levels were measured at 11 dpi. Data are means ± SE of replicates from three independent experiments, and significance was analyzed by the t-test: *P < 0.05, **P < 0.01. n.s.: not significant (TIF) [file pone.0212233.s006.tif]
